# Nurses’ perspectives on perioperative pulmonary rehabilitation for patients with lung cancer in the pre-implementation phase: a CFIR 2.0-guided study

**DOI:** 10.3389/fmed.2026.1904755

**Published:** 2026-10-05

**Authors:** Chunxiao Yang, Yi Liu, Fanfan Li, Chunmei Tan, Xiaoqiong Liu, Wenping Gong, Shirong Ma

**Affiliations:** 1 Department of Thoracic Surgery, Chongqing University Three Gorges Hospital, School of Medicine, Chongqing University, Chongqing, China; 2 Chongqing University Affiliated Renji Hospital (The Fifth People’s Hospital of Chongqing), School of Medicine, Chongqing University, Chongqing, China

**Keywords:** lung cancer, perioperative nursing, pulmonary rehabilitation, qualitative study, role heterogeneity, the updated Consolidated Framework for Implementation Research

## Abstract

**Background:**

Despite strong evidence supporting perioperative pulmonary rehabilitation (PR) for patients undergoing lung cancer surgery, little is known about factors that may influence implementation prior to program rollout, particularly in resource-constrained settings. Guided by the updated Consolidated Framework for Implementation Research (CFIR 2.0), this study aimed to identify anticipated barriers and facilitators to inform program refinement and future implementation strategies.

**Methods:**

A descriptive qualitative design was used. Semi-structured interviews were conducted with nurses from the thoracic surgery department of a tertiary hospital in China (September–November 2025). Guided by the four CFIR 2.0 domains (innovation, outer setting, inner setting, individuals), directed content analysis was performed based on nurses’ perspectives.

**Results:**

Sixteen nurses were interviewed, representing diverse roles: Ward/ICU frontline staff nurses, charge nurses, unit secretaries, administrative nurses and nurse managers. Relevant constructs included 22 of the 29 CFIR 2.0 constructs and 8 subconstructs. Facilitators spanned: innovation (source, evidence base, relative advantage, trialability); outer setting (financing, policy, external pressure); inner setting (relational connections, communication, culture, incentive systems); and individual factors (team needs, leadership/practitioner capability, motivation). Barriers comprised: innovation (adaptability, complexity, cost); outer setting (local condition, partnerships and connections); inner setting (structural characteristics, compatibility, access to knowledge and information); and individuals (recipient needs, capability). Notably, implementation concerns differed across nursing roles, reflecting role heterogeneity and underscoring the need for tailored implementation strategies.

**Conclusion:**

Implementation of perioperative PR for patients undergoing lung cancer surgery is influenced by multilevel factors spanning innovation, outer setting, inner setting, and individuals determinants. Differences in concerns across nursing roles highlight the importance of role-tailored implementation strategies. These findings provide a foundation for optimizing implementation approaches and may facilitate the sustainable integration of evidence-based PR into routine practice, particularly in resource-constrained healthcare settings.

## 1 Introduction

Lung cancer remains one of the most common and lethal malignancies worldwide, with an estimated 2.5 million new cases and 1.8 million deaths in 2022 (1). In China, more than 1 million new cases and over 700,000 lung cancer-related deaths were reported in the same year, making lung cancer the leading cause of cancer-related mortality nationwide (2). Although surgical resection remains the primary curative treatment for lung cancer, procedure-related lung tissue injury and postoperative deterioration in pulmonary function are often unavoidable. These adverse effects can significantly impair patients’ quality of life and limit their ability to perform daily activities following surgery (3, 4).

In developing countries, the implementation of evidence-based interventions is often constrained by limited healthcare resources, particularly in district- and county-level hospitals (5). These institutions serve a substantial proportion of the population but frequently face nursing workforce shortages, inadequate rehabilitation equipment, underdeveloped health information systems, and limited access to specialized professional training (6, 7). Furthermore, the Diagnosis-Related Groups (DRGs)-based payment system in China places increasing pressure on hospitals to shorten hospital stays, which may limit investment in rehabilitation programs requiring sustained time and personnel resources (8). Collectively, these challenges impede the integration of high-quality evidence into routine clinical practice and underscore the need for implementation research that is responsive to local resource constraints.

Among the evidence-based interventions recommended for patients undergoing lung cancer surgery, perioperative pulmonary rehabilitation (PR) has emerged as a key component of Enhanced Recovery After Surgery (ERAS) pathways and has received increasing attention in recent years (9–11). Perioperative PR is an individualized, multidisciplinary intervention delivered across the surgical continuum, with a core focus on preoperative prehabilitation and early postoperative rehabilitation. It encompasses a range of non-pharmacological strategies, including aerobic and resistance exercise training, breathing retraining, patient education on disease management and self-care, nutritional support, and psychological counseling (12–14). The primary objectives of perioperative PR are to improve respiratory function, physical capacity, and psychological wellbeing, thereby reducing the incidence of postoperative pulmonary complications, accelerating postoperative recovery, and enhancing health-related quality of life (15). Accordingly, a growing body of evidence and several evidence-based clinical guidelines recommend integrating PR throughout the perioperative management of patients undergoing lung cancer surgery (16–18).

Despite these recommendations, the implementation of perioperative PR in routine clinical practice remains limited. Existing studies have primarily focused on single-component interventions or on specific perioperative phases, such as the preoperative or postoperative period (19–21). Consequently, evidence regarding the delivery and management of comprehensive perioperative PR programs remains insufficient. Moreover, current knowledge is derived predominantly from evidence syntheses, including systematic reviews, clinical practice guidelines, and evidence summaries, whereas studies examining how evidence-based PR programs can be implemented in real-world clinical settings remain relatively scarce (22–25). To address this gap, our research team systematically synthesized relevant guidelines and evidence summaries published over the past 3 years (24–26) and developed a preliminary perioperative PR management program for patients undergoing lung cancer surgery. However, the feasibility and implementation of this program may be influenced by contextual factors within healthcare settings, particularly where resources are constrained (27–29). The updated Consolidated Framework for Implementation Research (CFIR 2.0) provides a comprehensive framework for identifying and understanding such implementation determinants across five domains: innovation, outer setting, inner setting, individuals, and process (30). It has been widely applied to identify factors that facilitate or hinder the implementation of evidence-based interventions across diverse healthcare settings (31–34).

Nurses play a central role in translating perioperative PR recommendations into routine clinical practice because they maintain continuous contact with patients and integrate PR-related activities into daily care, including patient education, respiratory training, symptom monitoring, postoperative mobilization, and multidisciplinary coordination (35). As frontline implementers, nurses may encounter distinct barriers and facilitators depending on their roles and responsibilities within the healthcare team. Exploring these role-specific perspectives can provide a more comprehensive understanding of implementation determinants and inform tailored implementation strategies and professional development in resource-constrained settings.

Guided by the CFIR 2.0, this descriptive qualitative study aimed to explore role-specific perceptions of anticipated barriers and facilitators to implementing a perioperative PR management program among nurses involved in the care of patients undergoing lung cancer surgery. Specifically, the study sought to capture perspectives from nurse managers, charge nurse, frontline staff nurse, unit secretary and administrative nurse. Understanding similarities and differences across these nursing roles prior to implementation may help inform tailored implementation strategies, enhance the feasibility and effectiveness of evidence-based PR interventions, and support their sustainable integration into routine clinical practice.

## 2 Materials and methods

### 2.1 Study design

This study used a descriptive qualitative research method (36), to explore thoracic surgery nurses’ pre-implementation perceptions of potential barriers and facilitators to a perioperative pulmonary rehabilitation program for patients undergoing lung cancer surgery within a resource-constrained setting, with a specific focus on how role heterogeneity shapes implementation perspectives across different nursing positions. This approach helps to understand nurses’ experiences and perspectives on new protocol practices, providing the most direct and fundamental answers to protocol revisions.

### 2.2 Background settings

The study was conducted in the thoracic surgery department of Chongqing University Three Gorges Hospital, a tertiary hospital in China. The department provides comprehensive surgical care for patients with lung cancer and other thoracic diseases and includes general thoracic wards and an intensive care unit. However, the department operates within a resource-constrained healthcare environment, with challenges related to staffing, rehabilitation equipment, information systems, and access to specialized professional training. At the time of the study, a nurse-led evidence-based perioperative PR program for patients undergoing lung cancer surgery had been developed based on a systematic synthesis of evidence, including clinical practice guidelines, evidence summaries, and expert consensus (24–26), but had not yet been routinely implemented. The preliminary program encompassed key components of perioperative rehabilitation, including patient eligibility, rehabilitation timing, multidisciplinary team responsibilities, preoperative assessment, preoperative and postoperative interventions, comprehensive rehabilitation management throughout the perioperative period, and outcome evaluation (Supplementary Table 1). Given the complexity of implementing evidence-based PR in routine clinical practice, particularly in resource-constrained settings, a pre-implementation assessment was undertaken to explore potential barriers and facilitators from the perspectives of different nurses. The findings were intended to inform refinement of the program and optimize its feasibility, acceptability, and applicability within the local clinical context.

### 2.3 Setting-participants

From September to November 2025, purposive sampling with maximum variation was used to recruit participants from the thoracic surgery department of Chongqing University Three Gorges Hospital, China. To capture a broad range of perspectives regarding implementation of the perioperative PR program, nurses occupying different professional roles, including nurse managers, clinical nurses, and administrative nurses, and with varying years of clinical experience were invited to participate.

Eligible participants were required to: (1) hold a valid registered nurse license; (2) be employed in the thoracic surgery department; and (3) have at least 1 year of work experience in thoracic surgery nursing. Nurses rotating through the department, as well as those undertaking residency training, advanced training, or clinical internships, were excluded.

Written and verbal consent was obtained from the interview subjects for this study.

### 2.4 Data collection

Data collection was conducted between September and November 2025, prior to implementation of the perioperative PR management program. Individual semi-structured, in-depth interviews were used to explore nurses’ perceptions of potential barriers and facilitators to program implementation. All interviews were conducted by a nurse researcher, who was not involved in the development of the PR program and had extensive experience in communication and narrative nursing. To minimize potential bias, the interviewer maintained a neutral position throughout the study. The interview guide was developed based on the CFIR 2.0 Interview Guide Tool^1^ and addressed four domains (Innovation, Outer Setting, Inner Setting, and Individuals), excluding the Process domain as this was a pre-implementation study—no planning, engagement, execution, or evaluation activities had yet taken place, so participants could not meaningfully report on them (30). The preliminary interview guide was pilot-tested with two nurses and subsequently reviewed by two experts in thoracic surgery nursing and qualitative research. Following iterative discussions and revisions informed by the pilot interviews and expert feedback, the final interview guide was established (Supplementary Tables 2, 3). Interviews were conducted in a quiet and private office within the thoracic surgery department and were audio-recorded with participants’ permission. During the interviews, the interviewer followed participants’ responses flexibly rather than adhering strictly to the interview guide, allowing emerging topics to be explored in greater depth. Each interview lasted approximately 30–50 min. Field notes were recorded immediately after each interview to capture contextual information and researchers’ reflections. Data collection ceased when no new codes or themes emerged and thematic saturation was considered achieved.

### 2.5 Data analysis

Data analysis was conducted concurrently with data collection. Within 24 h of each interview, two trained researchers transcribed the audio recordings verbatim and integrated the transcripts with field notes and contextual observations. Following participant confirmation, the two researchers independently reviewed the transcripts to achieve data familiarization and then coded, managed, and analyzed the data using NVivo software (version 14.0). A directed content analysis approach guided by the CFIR 2.0 was employed, with coding and construct mapping informed by the CFIR 2.0 and its operational definitions. Initial coding was performed independently by the two researchers, after which codes were compared, discussed, and refined through an iterative process. Any discrepancies were resolved through team discussion until consensus was reached. The finalized codes were subsequently organized into categories and mapped to relevant CFIR 2.0 domains and constructs to identify perceived barriers and facilitators to implementation. To enhance the trustworthiness of the findings, several strategies were employed. Member checking was conducted by returning transcripts to participants for verification. Investigator triangulation was achieved through independent coding and analysis by two researchers, followed by regular peer debriefing and consensus discussions to resolve discrepancies. In addition, an audit trail documenting coding decisions and analytical processes was maintained to ensure transparency and dependability throughout the study.

Additionally, through the interviews, we quantified the total number of barriers and facilitators encountered when implementing the project according to the perceptions of the 16 participants.

### 2.6 Ethical considerations

This study was approved by the Institutional Review Board of the Chongqing University Three Gorges Hospital (IRB No. 2025022). All participants provided written informed consent before the interviews. Participation was voluntary, and participants were informed of their right to withdraw from the study at any time without consequences. Confidentiality was maintained throughout the study, and all interview data were de-identified and used solely for research purposes.

## 3 Results

### 3.1 Participant characteristics

A total of 16 nurses were interviewed, and participants were assigned identification codes ranging from XW1 to XW16 to ensure confidentiality. Participants ranged in age from 26 to 44 years and had between 2 and 25 years of nursing experience. To capture diverse perspectives on the implementation of the perioperative PR program, the sample included nurse managers, (ICU/Ward) charge nurse, frontline staff nurse, unit secretary and administrative nurse. Detailed demographic and professional characteristics of the participants are presented in Supplementary Table 4.

### 3.2 Barriers and facilitators

Among the 29 constructs in the 4 domains of the CFIR 2.0, 22 constructs and 8 subconstructs were found to be relevant to perioperative PR program for lung cancer patients. The specific facilitators and barriers are shown in Supplementary Table 5.

#### 3.2.1 Theme 1: Innovation

Several factors within the innovation domain were identified as facilitators for implementing the perioperative PR program for patients with lung cancer. More than half of the participants (*n* = 11) stated that they felt more confident about implementing the program when it was developed by a reliable team. Half of the participants (*n* = 8) indicated that a program based on current evidence would provide clear advantages for implementation. Most participants (*n* = 14) perceived the program as more systematic and comprehensive than existing rehabilitation measures. In addition, nearly all participants (*n* = 15) highlighted that the program’s trialability reinforced their perception of its safety.

*XW14: The team has experienced doctors and nurses in the department who are very professional in clinical diagnosis and nursing management… these measures come from evidence summaries and guidelines, and it can be seen that they are evidence-based, so I trust this team. The program is more comprehensive than the current one and is still friendly to novice nurses.*

*XW9: Of course, it can be piloted. This solution is still very safe. First select a few young patients with cooperative family members to try it out. The pilot also gives us a buffer period, so do not ask everyone to do it right away.*

Conversely, several participants (*n* = 11) expressed concerns regarding the applicability and time requirements of certain components of the program. Most participants (*n* = 12) also noted that the program involves multiple interconnected steps and that the evaluation scales are cumbersome, contributing to the overall complexity of the innovation.

*XW8: We cannot meet the standards for PR 2 to 4 weeks before surgery. Now that the examinations are mutually recognized, the surgery may be performed the next day after admission at the fastest, and within a week at the slowest. I do not think it should be so rigid.*

*XW4: The most important thing is that if one person does all these contents, time issues may lead to overtime work, and it is not enough to work overtime every day.*

*XW12: It’s just too much to fill out these forms. I not only have to write down nursing records, but I also have to write down these indicator collections.*

#### 3.2.2 Theme 2: Outer setting

Several outer factors were identified as facilitators for implementing the perioperative PR program for patients with lung cancer. A small number of participants (*n* = 4) indicated that support from additional funding projects and associated performance incentives could enhance nurses’ motivation to implement the program. More than half of the participants (*n* = 10) perceived that perioperative PR implementation is necessary and should be driven by policies such as Enhanced Recovery After Surgery (ERAS) and DRGs medical insurance. In addition, a small number of participants (*n* = 4) highlighted that pressure from advanced pulmonary rehabilitation practices in higher-level hospitals encouraged the adoption of similar approaches locally.

*XW5: The promotion of accelerated recovery, the national policy DRGs medical insurance policy, and the reduction of patient treatment costs all make me think that PR must be implemented.*

*XW1: XX Hospital is conducting ERAS related to sensitive indicators related to PR. Our hospital is also learning, and it must be done.*

*XW15: The department now has funds from the Chongqing Regional Early Lung Cancer Diagnosis and Treatment Center to help us study abroad and purchase equipment. If there are more such funding projects, I think our supporting facilities will definitely be more complete.*

Conversely, more than half of the participants (*n* = 10) expressed concerns that the absence of external support could hinder program implementation. A small number of participants (*n* = 4) also noted that the local economic context might influence decision-making by patients and their families.

*XW11: Without other external assistance, we can only explore it ourselves, which may be slow and encounter some problems in the process.*

*XW16: This has something to do with cost. Compared with the population in big cities, our cultural level and economic conditions are much lower. Patients and their families do not understand the actual usefulness of PR and are also worried about the cost, so the acceptance is poor.*

#### 3.2.3 Theme 3: Inner setting

Several factors within the hospital and thoracic surgery department were identified as facilitators of perioperative pulmonary rehabilitation (PR) program implementation. First, all participants (*n* = 16) emphasized that effective communication within and across departments, together with strong interdisciplinary collaboration, created a supportive environment for implementing the PR program. Second, nearly all participants (*n* = 14) reported that healthcare professionals, patients, and family members shared a common goal of promoting rapid recovery, which facilitated program implementation. In addition, more than half of the participants (*n* = 10) indicated that both financial incentives and professional recognition could enhance staff motivation and engagement in implementation activities.

*XW16: The communication within the department is excellent… Moreover, other departments provide substantial support to our department… This enhances our professional standing and also facilitates better cooperation from patients.*

*XW7: I hope that the patient will recover soon, the hospital stay will be shortened, and the hospital’s indicators will be met. Overall, it will be beneficial to the patient and us…*

*XW2: Nurses who receive positive feedback from patients regarding PR prior to the patients’ discharge shall be given comprehensive commendations, performance bonuses or small monetary rewards.*

Conversely, all participants (*n* = 16) identified limitations in organizational infrastructure and resources as barriers to implementation. These included inadequate physical resources and equipment (*n* = 7), insufficient information and management systems (*n* = 7), and suboptimal staffing arrangements (*n* = 14), all of which were perceived to increase workload and implementation burden. Furthermore, most participants (*n* = 10) reported that poor compatibility between the PR program and existing clinical workflows could hinder implementation. A small number of participants (*n* = 5) also noted that, although internal training opportunities were available, access to regional or national academic conferences was limited, thereby restricting opportunities for professional development and knowledge acquisition.

*XW1: An evaluation form that dynamically monitors the patient’s breathing training, like a body temperature sheet, I can know its continuous effect, otherwise it will be done blindly… I hope to support sufficient manpower arrangements, because health education is very time-consuming.*

*XW3: It includes some time allocation issues, such as our breathing training and exercise training. The time allocation may be crowded. Because there are other treatments in our department, they may conflict with other treatments.*

*XW10: I do not have a high degree of education and have few opportunities to participate in high-level academic conferences. I would very much like to have more learning opportunities.*

#### 3.2.4 Theme 4: Individuals

Several individual characteristics were identified as influential in the successful implementation of the perioperative PR program, including those of program recipients (patients and families), implementation team members (nurses), and departmental leaders. A subset of participants (*n* = 7) highlighted that aligning workload and compensation with the responsibilities of frontline staff could ensure motivation for program implementation. In addition, all participants (*n* = 16) perceived that implementation team members and leaders possessed strong communication skills, knowledge, and technical competence, which facilitated the smooth delivery of the PR program. Most participants (*n* = 8) also reported that nurses’ intrinsic motivation, combined with multi-faceted support, served as a primary driver for successful implementation.

*XW4: The department is not very busy at the moment. For now, my workload matches that of the income division. It will not affect the implementation of the plan, and it will not be a hindrance anyway.*

*XW6: I have very good clinical practice skills and am willing to actively cooperate. The nurse manager will definitely lead others to implement it. No one dares to refuse the leader’s order. Her communication skills will help promote our PR program.*

*XW10: See the positive feedback from patients. When the patient tells me, ‘cough according to the method you told me, the phlegm will come out easily and it will be much more comfortable’. This is the biggest motivation for me to persist.*

Conversely, several barriers related to program recipients were identified. Most participants (*n* = 13) noted that low health literacy among patients and families significantly impeded adherence to the PR program. Furthermore, most participants (*n* = 10) indicated that recipients’ needs for adequate analgesia were not consistently met, representing an important obstacle to effective program implementation.

*XW8: We are a district and county-level hospital. Compared with the residents of big cities, the overall educational level is relatively low, and most of them are elderly, so the acceptance ability is definitely worse than that of big cities. Patients may not follow the instructions taught by the nurse.*

*XW13: Some patients will refuse cough training because of fear of pain, which requires repeated communication and increases the difficulty of implementation.*

## 4 Discussion

This study provides important insights into the contextual factors that may influence the implementation of perioperative PR in resource-constrained healthcare settings. Unlike previous studies focusing primarily on intervention effectiveness or post-implementation experiences, this study identified implementation determinants before program rollout. The findings offer a foundation for developing tailored implementation strategies and optimizing the integration of evidence-based PR into routine clinical practice.

### 4.1 Innovation: balancing evidence-based strengths and practical feasibility

This study revealed a dual perception of the perioperative pulmonary rehabilitation (PR) program among nurses. On the one hand, most participants recognized the program’s strong evidence base, the credibility of the development team, and its potential advantages over existing rehabilitation practices. Participants frequently described the program as more systematic and comprehensive than current approaches and valued its trialability, which enhanced their confidence in its safety and feasibility. These findings are consistent with previous implementation research demonstrating that interventions supported by robust evidence and developed by trusted teams are more likely to be accepted and adopted by healthcare professionals (30).

On the other hand, more than half of the participants expressed concerns regarding the complexity and time demands associated with implementation. These concerns were particularly prominent among frontline nurses and nursing team leaders, who perceived the additional assessment, documentation, and intervention requirements as potentially burdensome within an already demanding workload. This finding highlights a persistent evidence–practice gap, whereby interventions supported by strong evidence may still encounter implementation challenges if they are not sufficiently aligned with the realities of clinical practice. It also underscores the importance of incorporating frontline practitioners’ perspectives during the intervention development and adaptation process.

A particularly important contextual factor is the increasingly shortened length of hospital stay for patients undergoing lung cancer surgery in China. Under policies promoting examination result sharing and the DRGs, some patients proceed from admission to surgery within only a few days (8, 53). As a result, implementing evidence-based recommendations that require several weeks of preoperative rehabilitation may be difficult in routine practice. Similar challenges have been reported in previous studies, which found that interventions often face resistance when they are not adequately adapted to organizational and healthcare system constraints (37). Therefore, future refinement of the PR program should prioritize adaptability while preserving its core evidence-based components. Potential strategies include developing differentiated rehabilitation pathways for patients with varying risk profiles and resource availability, simplifying assessment procedures, and integrating data collection into existing nursing documentation systems to minimize implementation burden and improve feasibility.

### 4.2 Outer setting: policy drivers vs. resource gaps

The promotion of ERAS principles and the implementation of DRGs policy were identified as important external drivers facilitating the implementation of the perioperative PR program (37, 52). Participants emphasized that national policies promoting accelerated recovery and cost-effective care reinforced the necessity of PR, with one nurse stating, “The promotion of accelerated recovery and the national DRGs to reduce patient treatment costs made me feel that PR must be implemented.” This aligns with previous research indicating that policy initiatives are among the strongest external motivators for change in clinical practice (38, 39). In addition, some nurses perceived technical pressure from higher-level hospitals, a “peer competition effect” that further heightened the sense of urgency for implementation.

Despite these macro-level policy drivers, only a minority of participants highlighted additional funding support or performance incentives as motivating factors. This suggests that, although external policies provide broad directional guidance, they have yet to be translated into concrete, perceptible incentives at the micro-level. Moreover, several participants noted that economic constraints, such as the current financial downturn, have reduced patients’ and families’ acceptance of PR, reflecting the influence of the socioeconomic environment on patient behavior—an aspect often overlooked in prior implementation research. Particularly in district- and county-level hospitals, patients’ financial capacity and health literacy significantly affect their willingness to engage in PR programs (40, 41). These findings underscore the need for differentiated education and support strategies that consider patients’ economic, social, and cultural contexts to enhance program adoption and effectiveness.

### 4.3 Inner setting: strong organizational culture amid resource structure constraints

The inner setting emerged as the domain with the highest concentration of both facilitators and barriers. All participants consistently emphasized the presence of smooth internal communication, effective interdisciplinary collaboration, and a shared commitment to “rapid recovery” among nurses, patients, and their families. This supportive organizational culture provides essential social capital for the implementation of the perioperative PR program. Consistent with prior studies (31, 42), effective team collaboration and leadership support serve as protective factors that alleviate work-related stress and enhance implementation confidence. Participants specifically highlighted that physicians were highly cooperative and departmental leaders actively advocated for nursing interests, suggesting that a supportive atmosphere may act as a buffer during program implementation by mitigating negative emotions associated with increased workload.

Despite this positive climate, structural deficiencies present significant challenges. Over two-thirds of participants indicated that “unreasonable staffing arrangements” increased workload, while more than one-third reported limitations in both hardware (e.g., dynamic monitoring devices) and software (e.g., hospital information systems). One nurse emphasized the importance of integrating digital monitoring into practice, noting the need for an evaluation tool to track patients’ respiratory training status, analogous to a temperature chart. Although recent domestic PR research has advanced toward digitalization and virtualization (43–45), most hospitals continue to rely on paper-based questionnaires for outcome evaluation, which limits continuous monitoring and constrains the assessment of intervention effectiveness.

Knowledge acquisition disparities further compound these challenges. Nurses with junior college education reported limited opportunities to attend high-level academic conferences, whereas frontline nurses with bachelor’s degrees had greater access to professional development through conferences and self-directed learning. This discrepancy not only reflects educational barriers but also reveals elements of professional marginalization, as nurses with lower educational attainment or those removed from direct clinical roles are more likely to be excluded from continuing education initiatives. To address these inequities, future internal training programs should proactively include marginalized staff to reduce hierarchical barriers and ensure equitable access to knowledge and skills.

### 4.4 Individuals: interacting influences of patients, nurses and leaders

Within the individuals domain, this study suggests that the implementation of perioperative PR is shaped by the characteristics and interactions of three key stakeholder groups: patients and their families, healthcare practitioners, and organizational leaders. Participants consistently identified patients’ and families’ understanding and acceptance of PR as critical determinants of adherence. This finding is highly consistent with previous studies demonstrating that health literacy and patient knowledge are important predictors of healthcare engagement and utilization (46, 47). Participants noted that many patients treated in district- and county-level hospitals are older adults with limited health literacy, which may reduce their understanding of and engagement with rehabilitation interventions. These findings suggest that implementation efforts should incorporate health education strategies tailored to individuals with varying literacy levels, such as video-based education, individualized instruction, and active family involvement.

With respect to healthcare practitioners, all participants emphasized that training could effectively address knowledge gaps and strengthen their confidence in delivering rehabilitation interventions. Participants generally reported a high level of self-efficacy, believing that their existing clinical skills were sufficient to support the implementation of PR. However, several nurses highlighted that inadequately managed postoperative pain could substantially hinder patient participation in rehabilitation activities. This finding reflects nurses’ strong concern for patient experience and is consistent with previous evidence indicating that effective pain management is a prerequisite for successful postoperative rehabilitation (48). Consequently, implementation of PR requires not only technical competence in rehabilitation delivery but also the ability to adapt communication approaches and intervention strategies according to patients’ evolving physical and emotional needs.

Leadership emerged as another influential factor. Nearly all participants perceived that leadership engagement, communication skills, and visible managerial support would enhance the priority and legitimacy of the PR program. Some participants suggested that managerial authority could facilitate compliance with implementation requirements; however, this observation also highlights a potential risk associated with relying primarily on hierarchical or directive approaches. Although such leadership patterns may reflect organizational realities within some healthcare settings (49), sustainable implementation is more likely to be achieved when external leadership support is accompanied by strong intrinsic motivation among frontline staff. Consistent with this perspective, participants frequently emphasized that observing positive patient outcomes and receiving favorable feedback were among the most meaningful sources of motivation for continued engagement in PR implementation. These findings suggest that future implementation strategies should balance leadership support with approaches that foster professional autonomy, value recognition, and a sense of personal accomplishment among nurses.

### 4.5 Differentiated concerns of nurses in different positions: from group analysis to role customization

An important finding of this study was the identification of role-specific concerns among nurses occupying different positions within the thoracic surgery department (50). Although participants were discussing the same perioperative PR program, the challenges they perceived varied considerably according to their professional responsibilities. Frontline nurses were primarily concerned with workload, staffing capacity, and time constraints; administrative nurses focused on documentation requirements and indicator reporting; nurses responsible for resource management emphasized material availability and associated costs; and nurse managers were more concerned with performance evaluation, coordination, and organizational support. These findings suggest that implementation challenges are not experienced uniformly across nursing roles.

This observation reflects a key strength of the CFIR 2.0, which distinguishes among implementation team members, innovation recipients, and leaders while also acknowledging heterogeneity within implementation teams themselves (30, 51). Accordingly, implementation strategies should move beyond a “one-size-fits-all” approach and instead be tailored to the specific needs and responsibilities of different professional roles. For example, optimizing staffing schedules may be particularly important for frontline nurses, whereas simplifying documentation procedures may better address the concerns of administrative personnel. In resource-constrained settings, role-tailored training and implementation support may be particularly important. Future strategies should therefore consider the practical needs of different nursing roles and promote cross-role communication and peer learning to facilitate collaborative problem-solving and sustainable implementation. Future implementation research should further examine role heterogeneity as a contextual factor influencing implementation outcomes, particularly in resource-limited healthcare settings.

The findings suggest that successful implementation of perioperative PR requires a multilevel implementation strategy. At the innovation level, intervention procedures should be simplified and adapted to local workflows. At the organizational level, staffing support and information-system integration should be strengthened. At the individual level, tailored educational approaches should be developed for both patients and healthcare professionals. These strategies may facilitate the sustainable integration of PR into routine thoracic surgical care, particularly in resource-constrained settings.

## 5 Conclusion

This study provides a comprehensive understanding of the multilevel factors that may influence the implementation of a perioperative PR program for patients undergoing lung cancer surgery. Guided by the CFIR 2.0, the findings demonstrate that implementation is shaped by a complex interplay of innovation characteristics, external policies, organizational context, and individual factors. While strong evidence support, favorable organizational culture, and leadership engagement facilitate implementation, challenges related to intervention complexity, resource limitations, patient health literacy, and workflow compatibility may hinder adoption. Importantly, the study highlights substantial role heterogeneity among nurses, indicating that implementation challenges and support needs vary across professional positions. These findings suggest that successful implementation of PR should move beyond uniform approaches and instead adopt tailored implementation strategies that account for contextual constraints and role-specific responsibilities.

### 5.1 Research limitation

The sample comes from a tertiary hospital, with limited generalizability. And there are only female nurses in the department, so the perspective of male nurses is not included.

### 5.2 Future research directions

By identifying implementation determinants before program rollout, this study provides an empirical foundation for optimizing implementation strategies and enhancing the feasibility, acceptability, and sustainability of evidence-based PR interventions. Future research should extend these findings through multicenter studies and longitudinal evaluations to examine how implementation determinants evolve across different phases of program adoption and sustainment.

## Data Availability

The raw data supporting the conclusions of this article will be made available by the authors, without undue reservation.

## Footnotes

1
https://cfirguide.org/
